# Supplementary material for: Heat adaptation of phage T7 under an extended genetic code
Source: Virus Evol. 2021 Dec 1;7(2):veab100. doi: 10.1093/ve/veab100 (PMC8923235; doi:10.1093/ve/veab100)
Supplement: veab100_Supp [file veab100_supp.zip › Sequencing_Analysis.zip!/Sequencing_Analysis/A1/evidence/SNP_4.html]

BRESEQ :: Evidence


| Predicted mutation | | | | | | |
| --- | --- | --- | --- | --- | --- | --- |
| evidence | seq id | position | mutation | annotation | gene | description |
| RA | NC\_001604.1 | 20,544 | G→A | intergenic (–/–) | – / – | –/– |

| Read alignment evidence... | | | | | | | | | | | |
| --- | --- | --- | --- | --- | --- | --- | --- | --- | --- | --- | --- |
|  | seq id | position | | ref | new | freq | score (cons/poly) | reads | annotation | genes | product |
| \* | NC\_001604.1 | 20,544 | 0 | G | A | 99.4% | 7076.7 / NA | 3205 | intergenic (–/–) | *–/–* | –/– |
| Reads supporting (aligned to +/- strand):  **ref** base G (5/5);  **major** base A (1550/1621);  **minor** base C (16/2);  **total** (1574/1631) | | | | | | | | | | | |
| Fisher's exact test for biased strand distribution *p*-value = 5.85e-04 | | | | | | | | | | | |
| Kolmogorov-Smirnov test that lower quality scores support variant *p*-value = 1.00e+00 | | | | | | | | | | | |

Only 100 of 3206 total aligned reads displayed.  

`GAGAAACGAACAGGACTTGCGGAGGATGGCGCAAAGTCTGTCTATGAGCGTTTAAAGAACGACCGTGCTCCCTATGAGACACGCGCTCAGAATTGCGCTCAATATACCATCCCATCATTGTTCCCTAAGGACTCCGATAACGCCTCTACAGATTATCAAACTCCGTGGCAAGCCGTGGGCGCTCGTGGTCTGAACAATCTAGCCTCTAAGCTCATGCTGGCTCTATTCCCTATGCAGACTTGGATGCGACTTACTATATCTGAATATGAAGCAAAGCAGTTACTGAGCGACCCCGATGGACTCGCTAAGGTCGATGAGGGCCTCTCGAT‑GGTAGAGCGTATCATCATGAACTACATTGAGTCTAACAGTTACCGCGTGACTCTCTTTGAGGCTCTCAAACAGTTAGTCGTAGCTGGTAACGTCCTGCTGTACCTACCGGAACCGGAAGGGTCAAACTATAATCCCATGAAGCTGTACCGATTGTCTTCTTATGTGGTCCAACGAGACGCATTCGGCAACGTTCTGCAAATGGTGACTCGTGACCAGATAGCTTTTGGTGCTCTCCCTGAGGACATCCGTAAGGCTGTAGAAGGTC  >  NC_001604.1/20246‑20840`  

                                                                                                                                                                                                                                                                                                          `|`                                                                                                                                                                                                                                                                                                           
`gagaAACGAACAGGACTTGCGGAGGATGGCGCAAAGTCTGTCTATGAGCGTTTAAAGAACGACCGTGCTCCCTATGCGACACGCGCTCAGAATTGCGCTCAATATACCATCCCATCATTGTTCCCTAAGGACTCCGATAACGCCTCTACAGATTATCAAACTCCGTGGCAAGCCTTGGGCGCTCTTCGTCTGAACAATCTATCCTCTAAGCTCATGCTGTCTCTATTCCCTATGCAGACTTTGATGCGACTTACTATTTCTGAATATGAAGCAAACCATTTACTGTGCGACCCCGATGAAc                                                                                                                                                                                                                                                                                                         >  1:83743/1‑301 (MQ=255)`  
  `aaaaCGAACAGGACTTGCGGAGGATGGCGAAAAGTCTGTCTATGAGCGTTTAAAGAACGACCGTGCTCCCTATGAGACACGCGCTCAGAATTGCGCTCAATATACCATCCCATCATTGTTCCCTAAGGACTCCGATAACGCCTCTACAGATTATCAAACTCCGTGGCAAGCCGTGGGCGCTCGTGGTCTGAACAATCTAGCCTCTAAGCTCATGCTGGCTCTATTCCCTATGCAGACTTGGATGCGACTTACTATATCTGAATATGAAGCAAAGCAGTTACTGAGCGACCCCGATGAACTc                                                                                                                                                                                                                                                                                                       <  1:112254/300‑1 (MQ=255)`  
          `cAGGACTTGCGGAGGATGGAGCAAAGTCTGTCTATGAGCGTTTAAAGAACGACCGTGCTCCCTATGAGACACGCGCTCAGAATTGCGCTAAATATACCAGCCCATCATTGTTCCCTAAGGACTCCGATAACGCCTCTACAGATTATCAAACTCCGTGGCAAGCCGTGGGCGCTCGTGGTCTGAACAATCTAGCCTCTAAGCTCATGCTGGCTCTATTCCCTATGCAGACTTGGATGCGACTTACTATATCTGAATATGAAGCAAAGCAGTTACTGAGCGACCCCGATGAACTCGCTAAGGt                                                                                                                                                                                                                                                                                               <  1:425898/301‑1 (MQ=255)`  
                          `tGGCGCAAAGTCTGTCTATGATCGTTTAAAGAACTACCGTGCTCCCTATGAGACACGCGCTCAGAATTGCGCTCAATATACCATCCCATCATTGTTCCCTAATGACTCCGATAACGCCTCTACAGATTATCAAACTCCGTGGCAAGCCGTGGGCGCTCGTGGTCTGAACAATCTAGCCTCTAAGCTCATGCTGGCTCTATTCCCTATGCAGACTTGGATGCGACTTACTATATCTGAATATGAAGCAAAGCAGTTACTGAGCGACCCCGATGAACTCGCTAAGGTCGATGAGGGCCTCTCg                                                                                                                                                                                                                                                                               <  1:271708/301‑1 (MQ=255)`  
                          `tGGCGCAAAGTATTTCTATGAGCGTTTAAAGAACGACCGTGCTCCCTATGAGACACGCGCTCAGAATTGCGCTCAATATACCATCCCATCATTGTTCCCTAAGGACTCCGATAACGCCTCTACAGATTATCAAACTCCGTGGCAAGCCGTGGGCGCTCGTGGTCTGAACAATCTAGCCTCTAAGCTCATGCTGGCTCTATTCCCTATGCAGACTTGGATGCGACTTACTATATCTGAATATGAAGCAAAGCAGTTACTGAGCGACCCCGATGAACTCGCTAAGGTCGATGAGGGCCTCTCg                                                                                                                                                                                                                                                                               <  1:132945/301‑1 (MQ=255)`  
                               `cAAAGTCTGTCTATGATCGTTTAAAGAACGACCGTGCTCCCTATGAGACACGCGCTCAGAATTGCGCTCAATATACCATCCCATCATTGTTCCCTAAGGACTCCGATAACGCCTCTACAGATTATCAAACTCCGTGGCAAGCCGTGGGCGCTCGTGGTCTGAACAATCTAGCCTCTAAGCTCATGCTGGCTCTATTCCCTATGCAGACTTGGATGCGACTTACTATATCTGAATATGAAGCAAAGCAGTTACTGAGCGACCCCGATGAACTCGCTAAGGTCGATGAGGGCCTCTCGAT‑GGt                                                                                                                                                                                                                                                                         <  1:287921/301‑1 (MQ=255)`  
                                     `ctgtctATGAGCGTTTAAAGAACGACCGTGCTCCCTATGAGACACGCGCTCAGAATTGCGCTCAATATACCATCCCATCATTGTTCCCTAAGGACTCCGATAACGCCTCTACAGATTATCAAACTCCGTGGCAAGCCGTGGGCGCTCGTGGTCTGAACAATCTATCCTCTAAGCTCATGCTGGCTCTATTCCCTATGCAGACTTGGATGCGACTTACTATATCTGAATATGAAGCAAAGCAGTTACTGAGCGACCCCGATGAACTCGCTAAGGTCGATGAGGGACTCTCGCT‑gtctcttat                                                                                                                                                                                                                                                                   >  1:420673/1‑293 (MQ=255)`  
                                      `tgtctATGAGCGTTTAAAGAACGACCGTGCTCCCTATGAGACACGCGCTCAGAATTGCGCTCAATATACCATCCCATCATTGTTCCCTAAGGACTCCGATAACGCCTCTACAGATTATCAAACTCCGTGGCAAGCCGTGGGCGCTCGTGGTCTGAACAATCTAGCCTCTAAGCTCATGCTGGCTCTATTCCCTATGCAGACTTGGATGCGACTTACTATATCTGAATATGAAGCAAAGCAGTTACTGAGCGACCCCGATGAACTCGCTAAGGTCGATGAGGGCCTCTCGAT‑GGTAGAGCGt                                                                                                                                                                                                                                                                  <  1:173900/301‑1 (MQ=255)`  
                                       `gtctATGAGCGTTTAAAGAACGACCGTGCTCCCTATGAGACACGCGCTCAGAATTGCGCTCAATATACCATCCCATCATTGTTCCCTAAGGACTCCGATAACGCCTCTACAGATTATCAAACTCCGTGGCAAGCCGTGGGCGCTCGTGGTCTGAACAATCTAGCCTCTAAGCTCATGCTGGCTCTATTCCCTATGCAGACTTGGATGCGACTTACTATATCTGAATATGAAGCAAAGCAGTTACTGAGCGACCCCGATGAACTCGCTAAGGTCGATGAGGCCCTCTCGAT‑GGTAGAGctgt                                                                                                                                                                                                                                                                 >  1:499854/1‑298 (MQ=255)`  
                                       `gtctATGAGCGTTTAAAGAACGACCGTGCTCCCTATGAGACACGCGCTCAGAATTGCGCTCAATATACCATCCCATCATTGTTCCCTAAGGACTCCGATAACGCCTCTACAGATTATCAAACTCCGTGGCAAGCCGTGGGCGCTCGTGGTCTGAACAATCTAGCCTCTAAGCTCATGCTGGCTCTATTCCCTATGCAGACTTGGATGCGACTTACTATACCTGAATATGAATCACAGCAGTTACTCAGCGACCCCGATGAACTCGCTAAGGTCGATGAGTGCCTCTCGAT‑GGTACAGCGtt                                                                                                                                                                                                                                                                 >  1:219537/1‑300 (MQ=255)`  
                                                `agTTTAAAGAACGACCGTGCTCCCTATGAGACACGCGCTCAGAATTGCGCTCAATATACCATCCCATCATTGTTCCCTAAGGACTCCGATAACGCCTCTACAGATTATCAAACTCCGTGGCAAGCCGTGGGCGCTCGTGGTCTGAACAATCTAGCCTCTAAGCTCATGCTGGCTCTATTCCCTATGCAGACTTGGATGCGACTTACTATATCTGAATATGAAGCAAAGCAGTTACTGAGCGACCCCGATGAACTCGCTAAGGTCGATGAGGGCCTCTCGAT‑GATAGAGCGTATCATCATGa                                                                                                                                                                                                                                                        >  1:444486/2‑301 (MQ=255)`  
                                                      `aaGAACGACCGTGCTCCCTATGAGACACGCGCTCAGAATTGCGCTCAATATACCATCCCATCATTGTTCCCTAAGGACTCCGATAACGCCTCTACAGATTATCAAACTCCGTGGCAAGCCGTGGGCGCTCGTGGTCTGAACAATCTAGCCTCTAAGCTCATGCTGGCTCTATTCCCTATGCAGACTTGGATGCGACTTACTATAACTGAATATGAAGCAAAGCAGTTACTGCGCGACCCCGATGAACTCGCTAAGGTCGATGAGGGCCTCTCGAT‑GGTATAGCGTATCATCATGAACTACa                                                                                                                                                                                                                                                  >  1:194014/1‑301 (MQ=255)`  
                                                       `aGAACGACCGTGCTCCCTATGAGACACGCGCTCAGAATTGCGCTCAATATACCATCCCATCATTGTTCCCTAATGACTCCGATAACGCCTCTACAGATTATCAAACTCCGTGGCAAGCCGTGGGCGCTCGTGGTCTGAACAATCTAGCCTCTAAGCTCATGCTGGCTCTATTCCCTATGCAGACTTGGATGCGACTTACTATATCTGAATATGAAGCAAAGCAGTTACTGAGCTACCCCGATGAACTCGCTAAGGTCGATGAGGGCCTCTCGAT‑GGTAGAGCGTATCATCATGAACTACAt                                                                                                                                                                                                                                                 <  1:331636/301‑1 (MQ=255)`  
                                                             `aCCGTGCTCCCTATGAGACACGCGCTCAGAATTGCGCTCAATATACCATCCCATCATTGTTCCCTAAGGACTCCGATAACGCCTCTACAGATTATCAAACTCCGTGGCAAGCCGTGGGCGCTCGTGGTCTGAACAATCTAGCCTCTCATCTCATGCTGGCTCTCTTCCCTATGCAGCCTTGGATGCGACTTACTATATCTGAATATGAATCAAAGCAGTTACTGAGCGACCCCGATTAACTCGCTAAGGTCGCTGAGGGCCTCTCGAT‑GGTAGAGCGTATCCTCATGAACTTCCTTGAGTc                                                                                                                                                                                                                                           >  1:255242/1‑301 (MQ=255)`  
                                                                  `gCTCCCTATGAGACACGCGCTCAGAATTGCGCTCAATATACCATCCCATCATTGTTCCCTAAGGACTCCGATAACGCCTCTACAGATTATCAAACTCCGTGGCAAGCCGTGGGCGCTCGTGGTCTGAACAATCTAGCCTCTAAGCTCATGCTGGCTCTATTCCCTATGCAGACTTGGATGCGACTTACTATATCTGAATATGAAGCAAAGCAGTTACTGAGCGACCCCGATGAACTCGCTAAGGTTGATGAGGGCCTCTCGAT‑GGTAGAGCGTATCATCATGAACTACATTGAGTCTAACa                                                                                                                                                                                                                                      >  1:361463/1‑301 (MQ=255)`  
                                                                   `aaagagacaggaCACGCGATCAGAATTGCGCTCAATATACCATCCCATCATTGTTCCCTAAGGACTCCGATAACGCCTCTACAGATTATCAAACTCCGTGGAAAGCCGTGGGCGCTCGTGGTTTGAACAATCTAGCCTCTAAGCTCATGCTGGCTCTATTCCCTATGCAGACTTGGATGCGACTTACTATATCTGAATATGAAGCAAAGCAGTTACTGAGCGACCCCGATGAACTCGCTAAGGTCGATGAGGGCCTCTCGAT‑GTTAGAGCGTATCATCATGAACTACATTGAGTCTAACAg                                                                                                                                                                                                                                     <  1:184647/291‑1 (MQ=255)`  
                                                                     `cccTATGAGACACGCGCTCAGAATTGCGCTCAATATACCATCCCATCATTGTTCCCTAAGGACTCCGATAACGCCTCTACAGATTATCAAACTCCGTGGCAAGCCGTGGGCGCTCGTGGTCTGAACAATCTAGCCTCTAAGCTCATGCTGGCTCTATTCCCTATGCAGACTTGGATGCGACTTACTATATCTGAATATGAAGCAAAGCAGTTACTGAGCGACCCCGATGAACTCGCTAATGTCGATGAGGGCCTCTCGAT‑GGTAGAGCGTATCATCATTAACTACATTTAGTCTAACAGtt                                                                                                                                                                                                                                   >  1:341091/1‑301 (MQ=255)`  
                                                                     `cccTATGAGACACGCGCTCAGAATTGCGCTCAATATACCATCCCATCATTGTTCCCTAAGGACTACGATAACGCCTCTACAGATTATCAAACTCCGTGGCCAGCCGTGGGCGCTCGTGGTCTGAACAATCTAGCCTCTCAGCTCATGCTGGCTCTATTCCCTATGCAGACTTGGATTCGACTTACTATATCTGAATATGAAGCAAACCAGTTACTGAACGACCCCGATGAATTCGCTAAGGTTGATGAGGCCCTCTGGTT‑GTTAGACCTTAACATCCTGAACTACttttgtgcaaaccgtt                                                                                                                                                                                                                                   >  1:507791/1‑288 (MQ=255)`  
                                                                       `ggtataagagaaagcTCAGAATTGCGCACAATATAACATCGCATCATTGTTCCCTAAGGACTCCGATAACGCCTATAAAGATAATCAAACACCGTGGCAATCCGTGGGCGATCGTAGTCTGAACAATCTAGCATATAAGCTGATGCTGGCTCTATTCACAATGCAGACTTGGATGCGACTTACTATATCTGAATATGAAGCAAAGCAATTACTGAGCGACCCCGAAGAACTCGCTAAGGTCGATGAGGGCCTCTAGAT‑GGTAGAGCGTATCATCATGAACTACATTGAGTCTAACAGTTAc                                                                                                                                                                                                                                 <  1:487041/288‑1 (MQ=255)`  
                                                                       `cgaggagaCACGGGCTAAGAATTGCGCACAAGATACCATCCCATCATTGGTCACTAAGGAATCCGATAACGCCTCTACAGATGATCAAACTCCGTGGCAAGCCGAGGGCGCTCGTGGTATGAACAATCTAGCCTATAAGCTCATGCTGGCTCTATTAACGAAGCAGACTGGGATGCGACTTACTATATCTGAATATGAAGCAAAGCAGTTACTGAGCGACCACGATGAAAACGCTAAGGTCGATGAGGGCCTCTAGAT‑GGTAGAGCGTATCATAATGAACTACATTGAGTCTAACAGTTAc                                                                                                                                                                                                                                 <  1:35314/297‑1 (MQ=255)`  
                                                                       `cTATGAGACACGCGCTCAGAATTGCGCTCAATATACCATCCCATCATTGTTCCCTAAGGACTCCGATAACGCCTCTACAGATTATCAAACTCCGTGGCAAGCCGTGGGCGCTCGTGGTCTGAACAATCTAGCCTCTAAGCTCATGCTGGCTCTATTCCCTATGCAGACTTGGATGCGACTTACTATATCTGAATATGAAGCAAAGCAGTTACTGAGCGACCCCGATGAACTCGCTAAGGTCGATGAGGGCCTCTCGAT‑GGTAGAGCGTATCATCATGAACTACCTTTAGTCTAACAGTTAc                                                                                                                                                                                                                                 >  1:386126/1‑301 (MQ=255)`  
                                                                             `gaCACGCGCTCAGAATTGCGCTAAATATACCATCCCATCATTGTTCCCTAAGTACTCCGATAACGCCTCTACAGATTATCAAACTCCGTGGCAAGCCGTGGGCGCTCGTGGTCTGAACAATCTAGCCTCTAAGCTCATGCTGGCTCTATTCCCTATGCAGACTTGGATGCGACTTACTATATCTGAATATGAAGCAAAGCAGTTACTGAGCGACCCCGATGAACTCGCTAAGGTCGATGAGGGCCTCTCGAT‑GGTAGAGAGTATCATCATGAACTACATTGAGTCTAACAGTTACCGCGTg                                                                                                                                                                                                                           <  1:66017/301‑1 (MQ=255)`  
                                                                                 `gagatgtgtataagagacagatatACCATCCCATCATTGTTCCCTAAGGACTCCGATAACGCCTCTACAGATTATCAAACTCCGTGGCAAGCCGTGGGCGCTCGTGGTCTGAACAATCTAGCCTCTAAGCTCATGCTGGCTCTATTCCCTATGCAGACTTGGATGCGACTTACTATATCTGAATATGAAGCAAAGCAGTTACTGAGCGACCCCGATGAACTCGCTAAGGTCGATGAGGGCCTCTCGAT‑GGTAGAGCGTATCATCATGAACTACATTGAGTCTAACAGTTACCGCGTGActc                                                                                                                                                                                                                       <  1:469023/281‑1 (MQ=255)`  
                                                                                      `tCAGAATTGCGCTCAATATACCATCCCATCATTGTTCCCTAAGGACTCCGATAACGCCTCTACAGATTATCAAACTCCGTGGCAAGCCGTGGGCGCTCGTGGTCTGAACAATCTAGCCTCTAAGCTCATGCTGGCTCTATTCCCTATGCAGACTTGGATGCGACTTACTATATCTGAATATGAAGCAAAGCAGTTACTGAGCGACCCCGATGAACTCGCTAAGGTCGATGATGGCCTCTCGAT‑GGTAGAGCGTATCATCATGAACTACATTGAGTCTAACAGTTACCGCGTGACTCTCttt                                                                                                                                                                                                                  <  1:151081/301‑1 (MQ=255)`  
                                                                                          `aaTTGCGCTCAATATACCATCCCATCATTGTTCCCTACGGACTCCGATAACGCCTCTACAGATTATCAAACTCCGTGGCAAGCCGTGGGCTCTCGTGGTCTGAACAATCTAGCCTCTAAGCTCATGCTGGCTCTATTCCCTATGCAGACTTGGATGCGACTTACTATATCTGAATATGAAGCAAAGCAGTTACTGAGCGACCCCGATGAACTCGCTAAGGTCGATGAGGGCCTCTAGAT‑GGTAGAGCGTATCATCATGAACTACATTGAGTCTAACAGTTACCACGTGACTCTCTTTGAgg                                                                                                                                                                                                              <  1:357146/301‑1 (MQ=255)`  
                                                                                                   `cAATATACCATCCCATCATTGTTCCCTAAGGACTCCGATAACGCCTCTACAGATTATCAAACTCCGTGGCAAGCCGTGGGCGCTCGTGGTCTGAACAATCTAGCCTCTAATCTCATGCTGGCTCTATTCCCTATGCAGACTTGGATGCGACTTACTATATCTGAATATGAAGCAAAGCAGTTACTGAGCGACCCCGATGAACTCGCTAAGGTCGATGAGGGCCTCTCGAT‑GGTAGAGCGTATCATCATGAACTACATTGAGTCTAACAGTTACCGCGTTACTCTCTTTGAGGCTCTCAAAc                                                                                                                                                                                                     >  1:430536/1‑301 (MQ=255)`  
                                                                                                   `cAATATACCATCCCATCATTGTTCCCTAAGGACTCCGATAACGCCTCTACAGATTATCAAACTCCGTGGCAAGCCGTGGGCGCTCGTGGTCTGAACAATCTAGCCTCTAAGCTCATGCTGGCTCTATTCCCTATGCAGACTTGGATGCGACTTACTATATCTGCATATGAAGCAAAGCAGTTACTGAGCGCCCCCTATGAACTCACTACGGTCTATGAGGTCCTCTCGAT‑TGTAGAGCGTATCATCATGTACTACATTGAGTCTAACAGTTACCGCGTTACACTCTTTGAgtcctgtctct                                                                                                                                                                                                     >  1:313464/1‑291 (MQ=255)`  
                                                                                                   `cAATATACCATCCCATCATTGTTCCCTAAGGACTCCGATAACGCCTCTACAGATTATCAAACTCCGTGGCAAGCCGTGGGCGCTCGTGGTCTGAACAATCTAGCCTCTAAGCTCATGCTGGCTCTATTCCCTATGCAGACTTGGATGCGACTTACTATATCTGAATATGAAGCAAAGCAGTTACTGAGCGACCCCGATGAACTCGCTAAGGTCGATGAGGGCCTCTCGAT‑GGTAGAGCGTATCATCATGAACTACATTGAGTCTAACAGTTACCGCGTGACTCTCTTTGAGGCTCTCAAAc                                                                                                                                                                                                     >  1:198522/1‑301 (MQ=255)`  
                                                                                                   `cAATATACCATCCCATCATTGTTCCCTAAGGACTCCGATAACGCCTCTACAGATTATCAAACTCCGTGGCAAGCCGTGGGCGCTCGTGGTCTGAACAATCTAGCCTCTAAGCTCATGCTGGCTCTATTCCCTATGCAGACTTGGATGCGACTTACTATATCTGAATATGAAGCAAAGCAGTTACTGAGCGACCCCGATGAACTCGCTAAGGTCGATGAGGGCCTCTCGAT‑GGTAGAGCGTATCATCATGAACTACATTGAGTCTAACAGTTACCGCGTGACTCTCTTTGAGGCTCTCAAAc                                                                                                                                                                                                     >  1:155730/1‑301 (MQ=255)`  
                                                                                                   `cAATATACCATCCCATCATTGTTCCCTAAGGACTCCGATAACGCCTCTACAGATTATCAAACTCCGTGGCAAGCCGTGGGCGCTCGTGGTCTGAACAATCTAGCCTCTAAGCTCATGCTGGCTCTATTCCCTATGCAAACTTGGATGCGACTTACTATATCTGAATATGAAGCAAAGCAGTTACTGAGCGACCCCGATGAACTCGCTAAGGTCGATGAGGGCCTCTCGAT‑GGTAGAGCGTATCATCATGAACTACATTGAGTCTAACAGTTACCGCGTGACTCTCTTTGAGGCTCTCAAAc                                                                                                                                                                                                     >  1:243094/1‑301 (MQ=255)`  
                                                                                                   `cAATATACCATCCCATCATTGTTCCCTAAGGACTCCGATAACGCCTCTACAGATTATCAAACTCCGTGGCAAGCCGTGGGCGCTCGTAGTCTGAGCAATCTAGCCTCTAAGCTCATGCTGGCTCTATTCCCTATGCAGACTTGGATGCGACTTACTATATCTGAATATGAAGCAAAGCAGTTACTGAGCGACCCCGATGAACTCGCTAAGGTCGATGAGGGCCTCTCGAT‑GGTAGAGCGTATCATCATGAACTACATTGAGTCTAACAGTTACCGCGTGACTCTCTTTGAGGCtgtctctt                                                                                                                                                                                                     >  1:164656/1‑297 (MQ=255)`  
                                                                                                   `cAATATACCATCCCATCATTGTTCCATAAGGACTCCGATAACGCCTCTACAGATTATCAAACTCCGTGGCAAGCCGTGGGCGCTCGTGGTCTGAACAATCTAGCCTCTAAGCTCATGCTGGCTCTATTCCCTATGCAGACTTGGATGCGACTTACTATATCTGAATATGAAGCAAAGCAGTTACTGAGCGACCCCGATGAACTCGCTAAGGTCGATGAGGGCCACTCGAT‑AGGAGAGCGTATCGTCATGAACTACATTGAGTCTAACAGTTACCGCGTGACTCTCTTTCAGGATCTCAAAc                                                                                                                                                                                                     >  1:208833/1‑301 (MQ=255)`  
                                                                                                   `aaagagacagtcccagcATTGTTCGCTAAGGACTACGATAACGCCTCTACAGATAATCAAACGCCTTGGCAAGCCGTGTGCGCTCGTGGTCTGAACAATCTAGCCTCTAAGCTCATGCTGGCTCTATTCCCTATGCAGACTTGGATGCGACTTACTATATCTGAATATGAAGCAAAGCAGTTACTGAGCGACCCCGATGAACTCGCTAAGGTCGATGAGGGCCTCTCGAT‑GGTAGAGCGTATCATCATGAACTACATGGAGTCTAACAGTTACCGCTTGACTCTCTTTGAGGCTCTCAAAc                                                                                                                                                                                                     <  1:411756/291‑1 (MQ=255)`  
                                                                                                            `atcccatcATTGTTCCCTAAGGACTCCGATAACGCCTCTACAGATTATCAAACTCCGTGGCAAGCCGTGGGCGCTCGTGGTCTGAACAATCTAGCCTCTAAGCTCATGCTGGCTCTATTCCCTATGCAGACTTGGATGCGACTTACTATATCTGAATATGAAGCAAAGCAGTTACTGAGCGACCCCGATGAACTCGCTAAGGTCGATGAGGGCCTCTCGAT‑GGTAGAGCGTATCATCATGAACTACATTTATTCTAACAGTTACCCCGTGACTCTCTTTGAGGCTCTCacaccgcggtctc                                                                                                                                                                                            >  1:491371/1‑292 (MQ=255)`  
                                                                                                             `tcccatcATTGTTCCCTAAGGACTCCGATAACGCCTCTACAGCTTATCAAACTCCGTGGCAAGCCGTTGGCGCTCGTGGTCTGAACAATCTAGCCTCTAAGCTAATGCTGGCTCTCTTCCCTATGCAGACTTGGATGCGACTTACTTTATCTGAATATGAAGCAAAGCAGTTACTGAGCGACCCCGATGAACTCGCTAAGGTCGATGAGGGCCTATCGAT‑GTTAGAGCTTATCATCATGAACTACATTGAGTTTAACAGTTACAGCGTGACTCTCTTTTAGACTCTCAACCCGTTCTTCGt                                                                                                                                                                                           >  1:146093/1‑301 (MQ=255)`  
                                                                                                             `tcccaacAGTGTTCCCGAAGGACTCCGATAACGCCTCTACAGATTATCAAACGCCGTGGCAAGCCGGGGGCGCGCGTGGTCTGAACAATCTAGCCTCTAAGCGCATGCTGGCTCTATTCCCTATGCAGACTTGGATGCGACTTACTATATCTGAATATGAAGCAAAGCAGTTACTGAGCGACCCCGATGAACTCGCTAAGGTCGATGAGGGCCTCTCGAT‑GGGAGAGCGTATCATCATGAACTACATTGAGTCTAACAGTTACCGCGTGACTCTCTTTGAGGCTCTCAAACAGTTAGTCGt                                                                                                                                                                                           <  1:440808/301‑1 (MQ=255)`  
                                                                                                                       `gTTCCCTAAGGACTCCGATAACGCCTCTACAGATTATCAAACTCCGTGGCAAGCCGTGGGCGCTCGTGGTCCGAACAATCTAGCCTCTAAGCTCATGCTGGCTCTATTCCCTATGCAGACTTGGATGCGACTTACTATATCTGAATATGAAGCAAAGCAGTTACTGAGCGACCCCGATGAACTCGCTAAGGTCGATGAGGGCCTCTCGCT‑GGTAGAGCGTATCATCATGAACTACATTGAGTCTAACCGTTACCGCGTGACTCTCTTTGAGGCTCTCAACCAGTTAGTCGTAGCTGGTaaa                                                                                                                                                                                 >  1:415308/1‑300 (MQ=255)`  
                                                                                                                         `tCCCTAAGGACTCCGATAACGCCTCTACAGATTATCAAACTCCGTGGCAAGCCGTGGGCGCTCGTGGTCTGAATAATCTAGCCTCTAAGCTCATGCTGGCTCTATTCCCTATGCAGACTTGGATGCGACTTACTATATCTGAATATGAAGCAAAGCAGTTACTGAGCGACCCCGATGAACTCGCTAAGGTCGATGAGGGCCTCTCGAT‑GGTAGAGCGTATCATCATGAACTACATTGAGTCTAACAGTTACCGCGTGACTCTCTTTGAGGCTCTCAAACAGTTAGTCGTAGCTGGTAAcct                                                                                                                                                                               >  1:233454/1‑299 (MQ=255)`  
                                                                                                                          `cccTAAGGACTCCGATAACGCACCTACAGATAATCAAAATCCGTTGCAAGTCGTGGGCGCTAGTTGTATGAACAATCTAGACTCTAAGCTCATGCTGGCTCTATTCCCTATGCAGACTTGGATGCGACGTACTAAATCTGAAGATGAAGGAAAGCAGTTAATGAGCTACCCCGATGAAAGCGCTAAGGTCGAAGAGGGCCTCTCGAT‑GGTAGAGCGTATCATCATGAACTACAATGAGTCTAACAGTTACCGCGTGACTCTCTTTGAGGCTCTCAAACAGTTAGTCGTAGCTGGTAACGTc                                                                                                                                                                              <  1:74491/301‑1 (MQ=255)`  
                                                                                                                           `catAAGGACTCCGATAACGCCTCTACAGATTATCAAACTCCGTGGCAAGCCGTGGGCGCTCGTGGTCTGAACAATCTAGCCTCTAAGCTCATGCTGGCTCTATTCCCTATGCAGACTTGGATGCGACTTACTATATCTGAATATGAAGCAAAGCAGTTACTGAGCGACCCCGATGAACTCGCTAAGGTCGATGAGGGCCTCTCGAT‑GGTAGAGCGTATCATCATGAACTACATTGAGTCTAACAGTTACCGCGTGACTCTCTTTGAGGCTCTCAAACAGTTAGTCGTAGCTGGTAACGTcc                                                                                                                                                                             <  1:100072/299‑1 (MQ=255)`  
                                                                                                                                   `cgccGATAACGCCTCGACAGATTATCAAACTCCGTGGCAAGCCGTGGGCGCTCGTGGTCTGAACAATCGAGCCTCTAAGCTCATGCTGGCTCTATGCCCTATGCAGACTTGTATGCGACTTACTATATCTGAATATGAAGCAAAGCAGTTACTGAGCGACCCCGATAAACTCGCTAAGGTCGATGAGGTCCTCTCGAT‑GGTAGAGCGTATCATCATGATCTACATTGAGTCTAACATTTACCGCTTGACTCTCTTTGAGTCTCTCAAACAGTTATTCGTAGCTGGTAACGTCCTGCTGtac                                                                                                                                                                     <  1:60346/299‑1 (MQ=255)`  
                                                                                                                                   `cTCCGATAACGCCTCTACAGATTATCAAACTCCGTGGCAAGCCGTGGGCGCTCGTGGTCTGAACAATCTAGCCTCTAAGCTCATGCTGGCTCTATTCCCTATGCAGACTTGGATGCGACTTACTATATCTGAATATGAAGCAAAGCAGTTACTGAGCGACCCCGATGAACTCGCTAAGGTCGATGAGGGCCTCTCGAT‑GGTAGAGCGTATCATCATGAACTACATTGAGTCTAACAGTTACCGCGTGACTCTCTTTGAGGCTCTCAAACAGTTAGTCGTAGCTGGTAACGTCCTGCTGtac                                                                                                                                                                     <  1:43597/301‑1 (MQ=255)`  
                                                                                                                                   `aagtgacagcGCCTCTACAGATTATCAAACTCCGTGGCAAGCCGTGGGCGCTCGTGGTCTGAACAATCTAGCCTCTAAGCTCATGCTGGCTCTATTCCCTATGCAGAATTGGATGCGACTTACTATATCTGAATATGAAGCAAAGCAGTTACTGAGCGACCCCGATGAACTCGCTAAGGTCGATGAGGGCCTCTCGAT‑GGTAGAGCGTATCATCATGAACTACATTGAGTCTAACAGTTACCGCGTGACTCTCTTTGAGGCTCTCAAACAGTTAGTCGTAGCTGGTAACGTCCTGCTGtac                                                                                                                                                                     <  1:283034/292‑1 (MQ=255)`  
                                                                                                                                    `cccGATAACGCCTCTACAGATTATCAAACTCCGTGGCAAGCCGTGGGCGCTCGTGGTCTGAACAATCTAGCCTCTAAGCTCATGCTGGCTCTATTCCCTATGCAGACTTGGATGCGACTTACTATATCTGAATATGAAGCAAAGCAGTTACTGAGCGACCCCGATGAACTCGCTAAGGTCGATGAGGGCCTCTCGAT‑GGTAGAGCGTATCATCATGAACTACATTGAGTCTAACAGTTACCGCGTGACTCTCTTTGAGGCTCTCAAACAGTTAGTCGTAGCTGGTAACGTCCTGCTGtacc                                                                                                                                                                    <  1:478697/300‑1 (MQ=255)`  
                                                                                                                                     `gctcggagatgtgtataagagacagaaCTCCGTGGCAAGCCGTGGGCGCTCGTGGTCTGAACAATCTAGCCTCTAAGCTCATGCTGGCTCTATTCCCTATGCAGACTTGGATGCGACTTACTATATCTGAATATGAAGCAAAGCAGTTACTGAGCGACCCCGATGAACTCGCTAAGGTCGATGAGGGCCTCTCGAT‑GGTAGAGCGTATCATCATGAACTACATTGAGTCTAACAGTTACCGCGTGACTCTCTTTGAGGCTCTCAAACAGTTAGTCGTAGCTGGTAACGTCCTGCTGtacct                                                                                                                                                                   <  1:29235/276‑1 (MQ=255)`  
                                                                                                                                       `gATAACGCCTCTACAGATTATCAAACTCCGTGGCAAGCCGTGGGCGCTCGTGGTCTGAACAATCTAGCCTCTAAGCTCATGCTGGCTCTATTCCCTATGCAGACTTGGATGCGACTTACTATATCTGAATATGAAGCAAAGCAGTTACTGAGCAACCCCGATGAACTCGCTAAGGTCGATGAGGGCCTCTCGAT‑GGTAGAGCGTATCATCATGAACTACATTGAGTCTAACAGTTACCGCGTGACTCTCTTTGAGGCTCTCAAACAGTTAGTCGTAGCTGGTAACGTCCTGCTGtacctac                                                                                                                                                                 >  1:267402/1‑301 (MQ=255)`  
                                                                                                                                        `aTAACGCCTCTACAGATTATCAAACTCCGTGGCAAGCCGTGGGCGCTCGTGGTCTGAACAATCTAGCCTCTAAGCTCATGCTGGATCTATTCCCTATGCAGACTTGGATGCGACTTACTATATCTGAATATGAAGCAAAGCAGTTACTGAGCGACCCCGATGAACTCGCTAAGGTCGATAAGGGCCTCTCGAT‑GGTAGAGCGTATCATCATGAACTACATTGAGTCTAACAGTTACCGCGTGACTCTCTTTGAGGCTCTCAAACAGTTAGTCGTAGCTGGTAACGTCCTGCTGtacctacc                                                                                                                                                                <  1:391640/301‑1 (MQ=255)`  
                                                                                                                                             `gCCTCTACAGATTATCAAACTCCGTGGCAAGCCGTGGGCGCTCGTGGTCTGAACAATCTAGCCTCTAAGCTCATGCTGGCTCTATTCCCTATGCAGACTTGGATGCGACTTACTATATCTGAATATGAAGCAAAGCAGTTACTGAGCGACCCCGATGAACTCGCTAAGGTCGATGAGGGCCTCTCGAT‑GGTAGAGCGTATCATCATGAACTACATTGAGTCTAACAGTTACCGCGTGACTCTCTTTGAGGCTCTCAAACAGTTAGTCGTAGCTGGTAACGTCCTGCTGTACCTaccggaac                                                                                                                                                           <  1:276158/301‑1 (MQ=255)`  
                                                                                                                                              `ccTCTACAGATTATCAAACTCCGTGGCAAGCCGTGGGCGCTCGTGGTCTGAACAATCTAGCCTCTAAGCTCATGCTGGCTCTATTCCCTATGCAGACTTGGATGCGACTTACTATATCTGAATATGAAGCAAAGCAGTTACTGAGCGACCCCGATGAACTCGCTAAGGTCGATGAGTGCCTCTCGAT‑GGTAGAGCGTATCATCATGAACTACATTGAGTCTAACAGTTACCGCGTGACTCTCTTTGAGGCTCTCAAACAGTTAGTCGTAGCTGGTAACGTCCTGCTGTCCCTaccggaacc                                                                                                                                                          >  1:510645/1‑301 (MQ=255)`  
                                                                                                                                                  `tACAGATTATCAACCTCCGTGGCAAGCCGTGGGCGCTCGTGGTCTGAACAATCTAGCCTATAAGCTCATGCTGGCTCTATTCCCTATGCAGACTTGGATGCGACTTACTATATCTTAATATTAAGCAAAGCAGTTACTGAGCGACCCCGATGAACTCGCTAAGGTCGATGAGTGCCTCTCGAT‑GGTAGAGCGTATCATCATGAACTACATTGAGTCTAACAGTTACCGCGTGACTCTCTTTGAGGCTCTCAAACAGTTAGTCGTAGCTGGTAACGTCCTGCTGTACATAccggaaccggaa                                                                                                                                                      <  1:451240/301‑1 (MQ=255)`  
                                                                                                                                                      `gATTATCAAACTCCGTGGCAAGCCGTGGGCGCTCGTGGTCTGAACAATCTAGCCTCTAAGCTCATGCTGGCTCTATTCCCTATGCAGACTTGGATGCGACTTACTATATCTGAATATGAAGCAAAGCAGTTACTGAGCGACCCCGATGAACTCGCTAAGGTCGATGAGGGCCTCTCGAT‑GGCAGAGCGTATCATCATGAACTACATTGAGTCTAAAAGTTACCGCGTGACTCTCTTTGTGGCTCTCAAACAGTTAGTCGTCGCTGGTAACGTCCTGCTGtacctgtctcttatacccatct                                                                                                                                                  >  1:105891/1‑284 (MQ=255)`  
                                                                                                                                                        `ttATCAAACTCCGTGGCAAGCCGTGGGCGCTCGTGGTCTGAACAATCTAGCCTCTAAGCTCATGCTGGCTCTATTCCCTATGCATACTTGGATGCGACTTACTATATCTGAATATGAAGCAAAGCAGTTACTGAGCGACCCCGATGAACTCGCTAAGGTCGATGAGGGCCTCTCGAT‑GGTAGAGCGTATCATCATGAACTACATTGAGTCTAACAGTTACCGCGTGACTCTCTTTGAGGCTCTCAAACAG‑TAGTCGTAGCTGGTAACGTCCTGCTGTACCTaccggaaccctgtctcttat                                                                                                                                               >  1:503531/1‑290 (MQ=255)`  
                                                                                                                                                        `ttATCAAACTCCGTGGCAAGCCGTGGGCGCTCGTGGTCTGAACAATCTAGCCTCTAAGCTCATGCTGGCTCTATTCCCTATGCAGACTTGGATGCGACTTACTATATCTGAATATGAAGCAAAGCAGTTACTGAGCGACCCCGATGAACTCGCTAAGGTCGATGAGGTCCTCTCGAT‑TGTAGAGCGTATCATCATGAACTACATTGAGTCTAACAGTTACCGCGTGACTCTCTTTGAGGCTCTCAAACAGTTAGTCGTAGCTGGTAACGTCCTGCTGtacctctgtctcttatacacatct                                                                                                                                                >  1:189652/1‑282 (MQ=255)`  
                                                                                                                                                        `ttATCAAACTCCGTGGCAAGCCGTGGGCGCTCGTGGTCTGAACAATCTAGCCTCTAAGCTCATGCTGGCTCTATTCCCTATGCAGACTTGGATGCGACTTACTATATCTGAATATGAAGCAAAGCAGTTACTGAGCGACCCCGATGAACTCGCTAAGGTCGATGAGGGCCTCTCGAT‑GGTAGAGCGTATCATCATGAACTACATTGAGTCTAACAGTTACCGCGTGACTCTCTTTGAGGCTCTCAAACAGTTAGTCGTAGCTGGTAACGTCCTGCTGTACCTaccggaacctgtctcttat                                                                                                                                                >  1:39652/1‑291 (MQ=255)`  
                                                                                                                                                        `ttATCAAACTCCGTGGCAAGCCGTGGGCGCTCGTGGTCTGAACAATCTAGCCTCTAAGCTCATGCTGGCTCTATTCCCTATGCAGACTTGGATGCGACTTACTATATCTGAATATGAAGCAAAGCAGTTACTGAGCGACCCCGATGAACTCGCTAAGGTCGATGAGGGCCTCTCGAT‑GGTAGAGCGTATCATCATGAACTACATTGAGTCTAACAGTTACCGCGTGACTCTCTTTGAGGCTCTCAAACAGTTAGTCGTAGCTGGTAACGTCCTGCTGTACCTACCGGCACCGGAAGGGTCa                                                                                                                                                >  1:307396/1‑301 (MQ=255)`  
                                                                                                                                                                 `cccGTGGCAAGCCGTGGGCGCTCGTGGTCTGAACAATCTAGCCTCTAAGCTCATGCTGGCTCTATTCCCTATGCAGACTTGGATGCGACTAACTATATCTGAATATGAAGCAAAGCAGTTACGGAGCGACCCCGATGAACTCGCTAAGGTCGATGAGGGCCTCTCGAT‑GGTAGAGCGTATCATCATGAACTACATTGAGTCTAACAGTTACCGCGTGACTCTCTTTGAGGCTCTCAAACAGTTAGTCGTAGCTGGTAACGTCCTGCTGTACCTACCGGAACCGGAAGGGTCAAACTATAAt                                                                                                                                       <  1:370327/300‑1 (MQ=255)`  
                                                                                                                                                                   `ggctcggagatgtgtataagagacagcTGAACAATCTAGCCTCTAAGCTCATGCTGGCTCTATTCCCTATGCAGACTTGGATGCGTCTTACTATATCTTAATATGAAGCAAAGCAGTTACTGAGCGACCCCCATGTACTCGCTAAGGTCGATGAGGGCCTCTCGAT‑GGTAGACCGTATCATCATCCACTACTTTGGGTCTAACAGTTACCGCGTGCCTCTCTTTGATGCTCTCAAACAGTTAGTCGTAGCTGGTAACGTCCTGCTGTACCTACCGGAACCGGAAGGGTCAAACTATAATcc                                                                                                                                     <  1:55301/275‑1 (MQ=255)`  
                                                                                                                                                                          `aGCCGTGGGCGCTCGTGGTTTGAACAAGCGAGCCTCTAAGCACATTCTCGCACTATCCCCTATGCAGACTTGGATGCGACTTACGATATCCGAATATGAAGCAAAGAAGTTACTGGGCGACGCCAAGAATCTCGCTAAGGTCTATGAGGGCCTCTCGAT‑GGTAGAGCGTATCATCATGAACTACATTGATTCTAACAGTTACCGCGGGACTCTCTTTGAGGCTCTCAAACAGTTAGTCGGAGCTGGTAACGTCCTGCTGTACCTACCGGAACCGGAAGGGTCAAACTATAATCCCATGAAg                                                                                                                              <  1:401588/301‑1 (MQ=255)`  
                                                                                                                                                                           `gCCGTGGGCGCTCGTGGTCTGAACAATCTAGCCTCTAAGCTCATGCTGGCTCTATTCCCTATGCAGACTTGGATGCGACTTACTATATCTGAATATGAAGCAAAGCAGTTACTGAGCGACCCCGATGAACTCGCTAAGGTCGATGAGGGCCTCTCGAT‑GGTAGAGCGTATCATCATGAACTACATTGAGTCTAACAGTTTCCGCGTGACTCTCTTTTAGGCTCTCAAACAGTTAATCGTAGCTGGTAACGTCCTGCTGTACCTACCGGAACCGGCAGGGTCCACCTTTACTccctgcttct                                                                                                                             >  1:474901/1‑294 (MQ=255)`  
                                                                                                                                                                                     `cTCGTGGTCTGAACAATCTAGCCTCTAAGCTCATGCTGGCTCTATTCCCTATGCAGACTTGGATGCGACTTACTATATCTGAATATGAAGCAAAGCAGTTACTGAGCGACCCCGATGAACTCGCTAAGGTCGATGAGGGCCTCTCGAT‑GGTAGAGCGTATAATCATGAACTACATTGAGTCTAACAGTTACCGCGTGACTCTCTTTGAGGCTCTCAAACAGTTAGTCGTATCTGGTAACGTCCTGCTGTACCTACCGGAACCGGAAGGGTGCAACTATAATccctttctcttaaccacatc                                                                                                                   >  1:120481/1‑284 (MQ=255)`  
                                                                                                                                                                                          `ggTCTGAACAATCTAGCCTCTAAGCTCATGCTGGCTCTATTCCCTATGCAGACTTGGATGCGACTTACTATATCTGAATATGAAGCAAAGCAGTTACTGAGCGACCCCGATGAACTCGCTAAGGTCGATGAGGGCCTCTCGAT‑GGTAGAGCGTATCATCATGAACTACATTGAGTCTAACAGTTACCGCGTGACTCTCTTTGAGGCTCTCAAACAGTTAGCCGTAGCTGGTAACGTCCTGCTGTACCTACCGGAACCGGAAGGGTCAAACTATAATTCCATGAAGCTGTACCGATTGtctt                                                                                                              >  1:336772/1‑301 (MQ=255)`  
                                                                                                                                                                                              `tataagcgacaGCCTCTAAGCTCATGCTGGCTCTATTCCCTATGCAGACGTGGATGCGACTTACTATATCTGAATATGAAGCAAAGCAGTTACTGAGCGACCCCGATGAACTCGCTAAGGTCGATGAGGGCCTCTCGAT‑GGTAGAGCGTATCATCATGAACTATATTGAGTCTAACAGTTACCACGTGACTCTCTTTGAGGCTCTCAAACAGTTAGTCGTAGCTGGTAACGTCCTGCTGTACCTACCGGAACCGGAAGGGTCAAACTATAATCCCATGAAGCTGTACCGATTGTCTTCTTa                                                                                                          <  1:238723/291‑1 (MQ=255)`  
                                                                                                                                                                                              `tGAACAATCTATCCTCTAAGCTCATGCTGGCTCTATTCCCTATGCAGACTTGTATGCGACTTACTATATCTGAATATGAAGCAAAGCAGTTACTGAGCTACCCCGATGAACACGCTAAGGTCTATGAGTGCCTATCGAT‑GGTAGAGCGTATCATCATGAACTACATTTAGTCAAACAGTTACCGCTTGACTCTCTTTGAGGCTCTCAAACAGTTAGTCGTAGCTGGTAACTTCCTGCTGTACCTACCGGAACCGGAAGGGTCAATCTATAATCCCATGAAGCTGTACCGATTGTCGTCTTa                                                                                                          <  1:141587/301‑1 (MQ=255)`  
                                                                                                                                                                                              `tGAACAATCTAGCCTCTAAGCTCATGCTGGCTCTATTCCCTATGCAGACTTGGATGCGACTTACTATATCCGAATATGAAGAAAAGCAGTTACTGAGCGACCCCGATGAACTCGCTAAGGTCGATGAGGGCCTCTCGAT‑GGTAGAGCGTATCATCATGAACTACATTGAGTCTAACAGTTACCGCGTGACTCTCTTTGAGGCTCTCAAACAGTTAGTCGTAGCTGGTAACGTCCTGCTGTACCTACCGGAACCGGAAGGGTCAAACTATAATCCCATGAAGCTGTACCGATTGTCTTCTTa                                                                                                          <  1:2632/301‑1 (MQ=255)`  
                                                                                                                                                                                               `gAACAATCTAGCCTCTAAGCTCATGCTGGCTCTATTCCATATGCAGACTTGGATGCGACTTACTATATCTGAATATGAAGCAAAGCAGTTACTGAGCGACCCCGATGAACTCGCTAAGGTCGATGAGGGCCTCTCGAT‑GGTAGAGCGTATCATCATGAACTACATTGAGTCTAACAGTTACCGCGTGACTCTCTTTGAGGCTCTCAAACAGTTAGTCGTAGCTGGTAACGTCCTGCTGTACCTACCGGAACCGGACGGGTCAAACTATAATCCCATGAAGCTGTACCGATTGTCTTCTTAt                                                                                                         >  1:29874/1‑301 (MQ=255)`  
                                                                                                                                                                                                `agcAATCTAGCCTCTAAGCTCATGCTGGCTCTATTCCCTATGCAGACTTGGATGCGACTTACTATATCTGAATATGAAGCAAAGCAGTTACTGAGCGACCCCGATGAACTCGCTAAGGTCGATGAGGGCCTCTCGAT‑GGTAGAGCGTATCATCATGAACTACATTGAGTCTAACAGTTACCGCGTGACTCTCTTTGAGGCTCTCAAACAGTTAGTCGTAGCTGGTAACGTCCTGCTGTACCTACCGGAACCGGAAGGGTCAAACTATAATCCCATGAAGCTGTACCGATTGTCTTCTTAtg                                                                                                        <  1:454810/299‑1 (MQ=255)`  
                                                                                                                                                                                                    `aTCTAGCCTCTAAGCTCATGCTGGCTCTATTCCCTATGCAGACTTGGATGCGACTTACTATATCTGAATATTAAGCAAAGCAGTTACTGAGCGACCCCGATGAACTCGCTAAGGTCGATGAGGGCCTCGCGAT‑GGTAGAGCGTATCATCATGAACTACATTGAGTCTAACAGTTACCGCGTGACTCTATTTGAGGCTCTCAAACAGTTAGTCCTAGCTGGTAACGTCCTGCTGTACCTACCGGAACCTGAAGGGTCAAAATATAATACCATGAAGCTGTACCGATTGTCTTCTTCTGTggg                                                                                                    >  1:279834/1‑300 (MQ=255)`  
                                                                                                                                                                                                    `aTCTAGCCTCTAAGCTCATGCTGGCTCTATTCCCTATGCAGACTTGGATGCGACTTACTATATCTGAATATGAAGCAAAGCAGTTACTGAGCGACCCCGATGAACTCGCTAAGGTCGATGAGGGCCTCTCGAT‑GGTAGAGCGTATCATCATGAACTACATTTAGTCTAACAGTTACCGCGTGACTCTCTTTGAGGCTCTCAAACAGTTAGTCGTAGCTGGTAACGTCCTGCTGTCCCTACCGGAACCGGAAGGGTCAAACTATAATCCCATGAAGCTGTACCGATTGTCTTCTTAtgctgt                                                                                                    >  1:349468/1‑297 (MQ=255)`  
                                                                                                                                                                                                             `ctAAGCTCATGCTGGCTCTATTCCCTATGCAGACTTGGATGCGACTTACTATATCTGAATATGAAGCAAAGCAGTTACTGAGCGACCCCGATGAACTCGCTAAGGTCGATGAGGGCCTCTCGAT‑GGTAGAGCGTATCATCATGAACTACATTGAGTCTAACAGTTACCGCGTGACTCTCTTTGAGGCTCTCAAACAGTTAGTCGTAGCTGGTAACGTCCTGCTGTACCTACCGGAACCGGAAGGGTCAAACTATAATCCCATGAAGCTGTCCCGATTGTCTTCTTATGTGGTCCAACgaga                                                                                           >  1:345613/1‑301 (MQ=255)`  
                                                                                                                                                                                                                `aggtCACGCTGGCTATATTCCGCATGCAGACTTGGAGGCGACTTACTAGATCAGAATACGAAGCAAAGCAGTGACTGAGCGACCCCGATGAACTCGCTAAGGGCGATGATGGCCTCTCGAT‑GGTAGAGCGTATCATCATGAACTACATGGAGTCTAACAGTTACCGCGTGACTCTCTTTGAGGCTCTCAAACAGTTAGTCGTAGCTGGTAACGTCCTGCTGTACCTACCGGAACCGGAAGGGTCAAACTATAATCCCATGAATCTGTACCGATTGTCTTCTTATGTGGTCCAACGAGACGc                                                                                        <  1:251595/298‑1 (MQ=255)`  
                                                                                                                                                                                                                 `gCTCATGCTGGCTCTATTCCCTATGCAGACTTGGATGCGACTTACTATATCTGAATATGAAGCAAAGCAGTTACTGAGCGACCCCGATGAACTCGCTAATGTCGATGAGGGCCTCTCGAT‑GGTAGAGCGTATCATCATGAACTACATTGAGTCTAACAGTTACCGCGTGACTCTCTTTGAGGCTCTCAAACAGTTAGTCGTAGCTGGTAACGTCCTGCTGTACCTACCGGAACCGGAAGGGTCAAACTATAATCCCATGAAGCAGTACCGATTGTCTTCTTAtgtgctgtctcttattcaa                                                                                       >  1:11589/1‑286 (MQ=255)`  
                                                                                                                                                                                                                 `gCTCATGCTGGCTCTATTCCCTATGCAGACTTGGATGCGACTTACTATATCTGAATATGAAGCAAAGCAGTTACTGAGCGACCCCGATGAACTCGCTAAGGTCGATGAGGGCCTCTCGAT‑TGTATAGAGTATCATCATGAACTACATTGAGTCTAACAGTTAACGCGTGACTTTCTTTGAGGCTCTCAAACAGTTAGTCGTAGCTGGTAACGTCCTGCTGTACCTCCCGGAAACGGAAGGGTCAAACTATAATCCCATGAAGATGTACCGATTGTCTTCTTATGTGGTCCAACgctgtctt                                                                                       >  1:93847/1‑294 (MQ=255)`  
                                                                                                                                                                                                                      `tGCTGGCTCTATTCCCTATGCAGACTTGGATGCGACTTACTATATCTGAATATGAAGCAAAGCAGTTACTGAGCGACCCCGATGAACTCGCTAAGGTCGATGAGGGCCTCTCGAT‑GGTAGAGCGTATCATCATGAACTACATTGAGTCTAACAGTTACCGCGTGACTCTCTTTGAGGCTCTCAAACAGTTAGTCGTAGCTGGTAACGTCCTGCTGTACCTACCGGAACCGGAAGGGTCAAACTATAATCCCATGAAGCTGTACCGATTGTCTTCTTATGTGGTCAAACGAGACGCATTCgg                                                                                  >  1:159265/1‑301 (MQ=255)`  
                                                                                                                                                                                                                       `gCTGTCTGTAGTCCCTATGCAGTCTTGGATGCGACTTACTATAACGAGATATGAAGCAACGGAGTTACTGAGCGACCCCGATGAACTCGGTAAGGTCTATGAGGGCCTCTCGAT‑GGTAGAGCGTATCATCATGAACTACATAGAGTCTAACAGATACCGCGTGACTCTCTTTGAGGCTCTCAAACAGTTAGTCGTAGCTGGTAACGTCCTGCTGTACCTACCGGAACCGGAAGGGTCAAACTATAATCCCATGAAGCTGTACCGATTGTCTTATTATGTGGTCCAACGAGACGCATTCGGc                                                                                 <  1:89423/301‑1 (MQ=255)`  
                                                                                                                                                                                                                          `ggCTCTATTCCCTATGCAGACTTTGATGCGACTTACTATATCTGAATATGAAGCAAAGCAGTTACTGAGCGACCCCGATGAACTCGCTAAGGTCGATGAGGGCCTCTCGAT‑TGTAGAGCGTATCATCATGAACTACATTGAGTCTAACAGTTACCGCGTGACTCTCTTTGAGGCTCTCAAACAGTTAGTCGTAGCTGGTAACGTCCTGCTGTACCTACCGGAACCGGAAGGGTCAAACTATAATCCCATGAAGCTGTACCGATTGTCTTCTTATGTGGTCCAACGAGACGCATTCGTCAAc                                                                              >  1:248420/1‑301 (MQ=255)`  
                                                                                                                                                                                                                                  `tCCCTATGCAGACTTGGATGCGACTTACTATATCTGAATATGAAGCAAAGCAGTTACTGAGCGACCCCGATGAACTCGCTAAGGTCGATGAGGGCCTCTCGAT‑GGTAGAGCGTATCATCATGAACTACATTGAGTCTAACAGTTACCGCGTGACTCTCTTTGAGGCTCTCAAACAGTTAGTCGTAGCTGGTAACGTCCTGCTGTACCTACCGGAACCGGAAGGGTCAAACTATAATCCCATGAAGCTGTACCGATTGTCTTCTTATGTGGTCCAACGAGACGCATTCGGCAGCGTTCTGCa                                                                      >  1:407944/1‑301 (MQ=255)`  
                                                                                                                                                                                                                                   `cccTAGGCAGACTTGGGAGCGACTTACTATATCTGAATATGAAGAAAAGCAGTTACTGAGCGACCCCGATGAACTAGCTAAGGTCGATGAGAGCCTCTCGAT‑GGTAGAGCGTATCATCATGAACTACATTGAGTCTAACAGGTACCGCGTGACTCTCTTTGAGGATCTCAAACAGTTAGTCGTAGCTGGTAACGTCCTGCTGTACCTACCGGAACCGGAAGGGTCAAACTATAATCCCATGAAGCTGTACCGATTGTCTTCTTATGTAGTCCAACGAGACGCATTCGGCAACGTTCTGCaa                                                                     <  1:365106/301‑1 (MQ=255)`  
                                                                                                                                                                                                                                       `aTGCAGACTTGGATGCGACTTACTATATCTGAATATGAAGCAAAGCAGTTACTGAGCGACCCCGATGAACTCGCTAAGGTCGATGAGGGCCTCTCGAT‑GGTAGAGCGTATCATCATGAACTACATTGAGTCTAACAGTTACCGCGTGACTCTCTTTGAGGCTCTCAAACAGTTAGTCGTAGCTGGTAACGTCCTGCTGTACCTATCGGAACCGGAAGGGTCAAACTATAATCCCATGAAGCTGTACCGATTGTCTTCTTATGTGGTCCAACGAGACGCATTCGGCAACGTTCTGCAAATgg                                                                 <  1:20463/301‑1 (MQ=255)`  
                                                                                                                                                                                                                                       `aTGCAGACTTGGATACGACTTACTATATCTGAATATGAAGCAAAGCAGTTACTGAGCGACCCCGAGGAACTCGCTAAGGTCGATGAGGGCCTCTCGAT‑GGTAGAGCGTATCATCATGAACTACATTGAGTCTAACAGTTACCGCTTGACTCTCTTTGAGGCTCTCAAACAGTTAGTCGTAGCTGGTAACGTCCTGCTGTACCTACCGGAACCGGAAGGGTCAAACTATAATCCCATGAAGCTGTACCGATTGTCTTCTTATGTGGTCCAACGAGACGCATTCGGCAACGTTCGGCAAATgg                                                                 <  1:464211/301‑1 (MQ=255)`  
                                                                                                                                                                                                                                                       `gACTTACTATATCTGAATATGAAGCAAAGCAGTTACTGAGCGACCCCGATGAACTCGATAAGGTCGATGAGGGCCTCTCGAT‑GGTAGAGCGTATCATCATGAACTACATTGAGTCTAACAGTTACCGCGTGACTCTCTTTGAGGCTCTCAAACAGTTAGTCGTAGCTGGTAACGTCCTGCTGTACCTACCGGAACCGGCAGGGTCAAACTATAATCCCATGACGCTGTACCGATTGTCTTCTTATGTGGTCCAACGAGACGCATTCAGCAACGTTCTGCAAATGGTGACTCGTGACCAGAt                                                 >  1:124055/1‑301 (MQ=255)`  
                                                                                                                                                                                                                                                           `tACTATATCTGAATATGAAGCAAAGCAGTTACTGAGCGACCCCGATGAACTCGCTAAGGTCGATGAGGGCCTCTCGAT‑GGTAGAGCGTATCATCATGAACTACATTGAGTCTAACAGTTACCGCGAGACTCTCTTTGAGGCTCTCAAACAGTTAGTCGTAGCTGGTAACGTCCTGCTGTACCTACCGGAACCGGAAGGGTCAAACTATAATCCCATGAAGCTGTACCGATTGTCTTCTTATGTGGTCCAACGAGACGCATTCGGCAACGTTCTGCAAATGGTGACTCGTGAccctgtctct                                             >  1:310415/1‑293 (MQ=255)`  
                                                                                                                                                                                                                                                           `tACTATATCTGAATATGAAGCAAAGCAGTTACTGAGCGACACCGATGAACTCGCTAAGGTCGATGACGGCCTCTCGAT‑GGTAGAGCGTATCATCATGAACTACATTGAGTCTAACAGTTACCGCGTGACTCTCTTTGAGGCTCTCAAACAGTTAGTCGTAGCTGGTAACGTCCTGCTGTACCTATCGGAACCGGAAGGGTCAAACTATAATCCCATGAAGCTGCACCGATTGTCTTCTTATGTGGTCCAACGAGACGCATTCGGCAACGTTCTGCAAATGGTGACTAGTGACCAGATAGCt                                             >  1:223808/1‑301 (MQ=255)`  
                                                                                                                                                                                                                                                            `aCTATATCTGAATATGAAGCAAAGCAGTTACTGAGCGACCCCGATGAACTCGCTAAGGTCGATGAGGGCCTCTCGAT‑GGTAGAGCGTATCATCATGAACTACATTGAGTCTAACAGTTACCGCGTGACTCTCTTTGAGGCTCTCAAACAGTTAGTCGTAGCTGGTAACGTCCTGCTGTACCTACCGGAACCGGAAGGGTCAAACTATAATCCCATGAAGCTGTACCGATTGTCTTCTTATGTGGTCCAACGAGACGCATTCGGCAACGTTCTGCAAATGGTGACTCGTGACCAGATAGCtt                                            >  1:127699/1‑301 (MQ=255)`  
                                                                                                                                                                                                                                                            `aCTATATCTGAATATGAAGCAAAGCAGTTACTGAGCGACCCCGATGAACTCGCTAAGGTCGATGAGGGCCTCTCGAT‑GGTAGAGCGTATCATCATGAACTACATTGAGTCTAACAGTTACCGCGTGACTCTCTTTGAGACTCTCAAACAGTTAGTCGTAGCTGGTAACGTCCTGCTGTACCTACCGGAACCGGAAGGGTCAAACTATAATCCCATGAAGCTGTACCGATTGTCTTCTTATGTGGTCCAACGAGACGCATTCGGCAACGTTCTGCAAATGGTGACTCGTGACCAGATAGCtt                                            >  1:214519/1‑301 (MQ=255)`  
                                                                                                                                                                                                                                                               `atatCTGAATATGAAGCAAAGCAGTTACTGATCGACCCCGATGAACTCGCTAAGGTCGATGAGGGCCTCTCGAT‑GGTAGAGCGTATCATCATGAACTACATTGAGTCTAACAGTTACCGCGTGACTCTCTTTGAGGCTCTCAAACAGTTAGTCGTAGCCGGTAACGTCCTGCTGTACCTACCGGAACCGGAAGGGTCAAACTATAATCCCATGAAGCTGTACCGATTGTCTTCTTATGTGGTCCAACGAGACGCATTCGGCAACGTTCTGCAAATGGTGACTCGTGACCAGATAGCtttct                                         >  1:495594/1‑299 (MQ=255)`  
                                                                                                                                                                                                                                                                `tatCTGAATAGGAAGCAAAGCAGTTACTGAGCGACCCCGATGAACTCGCTAAGGTCGATGAGGGCCTCTCGAT‑GGTAGAGCGTATCATCATGAACTACATTGAGTCTAACAGTTACCGCGTGACTCTCTTTGAGGCTCTCAAACAGTTAGTCGTAGCTGGTAATGTCCTGCTGTACCTACCGGAACCGGAAGGGTCAAACTATAATCCCATGAAGCTGTACCGATTGTCTTCTTATGTGGTCCAACGAGACGCATTCGGCAACGTTCTGCAAATGGTGACTCGTGACCAGATAGCTTTTgg                                        <  1:228325/301‑1 (MQ=255)`  
                                                                                                                                                                                                                                                                 `atgtgtataagagacagagcaGTTACTGAGCGACCCAGATGAACTCGCGAAGGTCGATGAGGTCCTCTCGAT‑GGTAGAGCTTATCATCATGAACTACATTGAGTCTAACAGTTACCGCGTGACTCTCTTTGAGGCTCTCAAACAGTTAGTCGTAGCTGGTAACGTCCTGCTGTACCTACCGGAACCGGAAGGGTCAAACTATAATCCCATGAAGCTGTACCGATTGTCTTCTTATGTGGTCCAACGAGACGCATTCGGCAACGTTCTGCAAATGGTGACTCGTGACCAGATAGCTTTTGGt                                       <  1:380659/284‑1 (MQ=255)`  
                                                                                                                                                                                                                                                                 `atCTGAATATGAAGCAAAGCAGTTACTGAGCGACCCCGATGAACTCGCTAAGGTAGATGAGGGCCTCTCGAT‑GGTACAGCGTATCATCATTAACTACATTTAGTATAACAGTTACCTCGTGACTCTCTTTGTGGCTCTCAATCCGTTATTCGTAGCTGGTAACGTCCTGCTATACCTACCCGAACCGCAACGGTCAAACTATAATGCCATGAAGCTGTCCCGATTTTTTTCTTATATGCTCCAACGAGACGCCTTCGGCACCGTTCTGCAAATGGTGACTCGTGACCAGCTAGCttttttt                                       >  1:48306/1‑298 (MQ=255)`  
                                                                                                                                                                                                                                                                     `gAATATGAAGCAAAGCAGTTACTGAGCTACCCCGATGAACTCGCTAAGGTCGATGAGGGCCTCTCGATGGGTAGAGCGTATCATCATGAACTACATTGAGTCTAACAGTTACCGCGTGACTCTCTTTGAGGCTCTCAAACAGTTAGTCGTAGCTGGTAACGTCCTGCTGTACCTACCGGAACCGGAAGGGTCAAACTATAATCCCATGAAGCTGTACCGATTGTCTTCTTATGTGGTCCAACGAGACGCATTCGGCAACGTTCTGCAAATGGTGACTCGTGACCAGATAGCTTTTGGTGct                                    >  1:317178/1‑301 (MQ=255)`  
                                                                                                                                                                                                                                                                        `tatGAAGCAAAGCAGGTACGGAGCGACCCCAATGAACTCGCTAAGGTCGATGAGGGTCTCTGGAT‑GGTAGAGGGTATCATCATGAACTACATTGAGTCTAACAGTTACCGCGTGACTCTCTGTGAGGCTCTCAAACAGTTAGTCGTAGCTGGTAACGTCCTGCTGTACCTACCGGAACCGGAAGGGTCAAACTATAATCCCATGAAGCTGTACCGATTGTCTTCTTATGTGGTCCAACGAGACGCATTCGGCAACGTTCTGCAAATGGTGACTCGTGACCAGATAGCTTTTGGTGCTCTcc                                <  1:321904/301‑1 (MQ=255)`  
                                                                                                                                                                                                                                                                            `aagcaaagcaGTTACTGAGCGACCCCGATGAACTCGCTAAGGTCGATGAGGGCCTCTCGAT‑GGTAGAGCGTATCATCATGAACTACATTGAGTCTAACAGTTACCGCGTGACTCTCTTTGAGGCTCTCAAACAGTTAGTCGTAGCTGGTAACGTCCTGCTGTACCTACCGGAACCGGAAGGGTCAAACTATAATCCCATGAAGCTGTACCGATTGTCTTCTTATGTGGTCCAACGAGACGCATTCGGCAACGTTCTGCAAATGGTGACTCGTGACCAGATAGCTTTTGGTGCTATcccagg                            >  1:136804/1‑298 (MQ=255)`  
                                                                                                                                                                                                                                                                              `tgtataagagacaggAGCGACCCCGATGAACTCGCTAAGGTCGATGAGGGCCTCTCGAT‑GGTAGAGCGTATCATCATGAACTACATTGAGTCTAACAGTTACCGCGTGACTCTCTTTGAGGCTCTCAAACAGTTAGTCGTAGCTGGTAACGTCCTGCTGTACCTACCGGAACCGGAAGGGTCAAACTATAATCCCATGAAGCTGTACCGATTGTCTTCTTATGTGGTCCAACGAGACGCATTCGGCAACGTTCTGCAAATGGTGACTCGTGACCAGATAGCTTTTGGTGCTATCCCTGAgg                          <  1:79052/287‑1 (MQ=255)`  
                                                                                                                                                                                                                                                                               `gagacaggTTACTGAGCGACCCCGATGAACTCGCTAAGGTCGATGAGGGCCTCTCGAT‑GGTAGAGCGTATCATAATGAACTACATTGAGTCTAACAGTTACCGCGTGACTCTCTTTGAGGCTCTCAAACAGTTAGTCGTAGCTGGTAACGTACTGCTGTACCTACCGGAACCGGAAGGGTCAAACTATAATCCCATGAAGCTGTACCGATTGTCTTCTTATGTGGTCCAACGAGACGCATTCGGCAACGTTCTGCAAATGGTGACTCGTGACCAGATAGCTTTTGGTGCTCTCACTGAGGa                         <  1:258888/294‑1 (MQ=255)`  
                                                                                                                                                                                                                                                                                `aaagcaGTTACTGAGCGACCCAGATGAACTCGCTAAGGTCGATGAGGGCCTCTCGAT‑GGTAGAGCGTATCATCATGAACTACATTGAGTCTAACAGTTACCGCGTGACTCTCTTTGAGGCTCTCAAACAGTTAGTCGTAGCTGGTAACGTCCTGCTGTACCTACCGGAACCGGAAGGGTCAAACTATAATCCCATGAAGCTGTACCGTTTGTCTTCTTATGTGGTCCAACGTGACGCATTCGGCAACGTTCTGCAAATGGTGACTCGTGACCAGATAGCTTTTGGTGCTCTCCCTGAGGAc                        >  1:459688/1‑301 (MQ=255)`  
                                                                                                                                                                                                                                                                                   `gcaGTTACTGAGCGACCCCGATGAACTCGCTAAGGTCGATGAGGTCCTCTCGAT‑GGTAGAGCGTATCATCATGAACTACATTGAGTCTAACAGTTACCGCGTGACTCTCTTTGAGGCTCTCAAACAGTTAGTCGTAGCTGGTAACGTCCTGCTGTACCTACCGGAACCGGAAGGGTCAAACTATAATCCCATGAAGCTGTACCGATTGTCTTCTTATGTGGTCCAACGAGACGCATTCGGCAATGTTCTGCAAATGGTGACTCGTGACCAGATAGCTTTTGGTGCTCTCCCTGAggcctgt                     >  1:179822/1‑296 (MQ=255)`  
                                                                                                                                                                                                                                                                                   `gcaGTAACTGCGCGACCCCGAGTAACTAGCTAAGGTCGAGGATGGCTTCTCGAT‑GGTAGAGCGTATCATCATGACCTACATTGAGTCTAACAGTTACCGCGTGACTCTCCTTGAGGCTCTCAAACAGTTAGTCGTAGCTGGTAACGTCCTGCTGTACCTACCGGATCCGGAAGTGTCAAACTATAATCCCATGAAGCTGTACCGATTGTCTTCTTATGTGGTCCAACGAGACGCATTCGGCAACGTTCTGCAAATGGTGACTCGTGACCAGATAGCTTTTGGTGCTCTACCTGAGGACATc                     <  1:326413/301‑1 (MQ=255)`  
                                                                                                                                                                                                                                                                                      `gTTACTGAGCGACCCCGATGAACTCGCTAAGGTCGATGAGGGCCTCTCGAT‑GGTAGAGCGTATCATCATGAACTACATTGAGTCTAACAGTTACCGCGTGACTCTCTTTGAGGATCTCAAACAGTTAGTCGTAGCTGGTAACGTCCTGCTGTACCTACCGGAACCGGAAGGGTCAAACTATAATACCATGAAGCTGTACCGATTGTCTTCTTATGTGGTCCAACGAGACGCATTCGGCAACGTTCTGCAAATGGTGACTCGTGACCAGATAGCTTTTGGTGCTCTCCCTGAGGACATCCGt                  >  1:169235/1‑301 (MQ=255)`  
                                                                                                                                                                                                                                                                                                `caCCCCGATTAACTCGTTAAGGTCTGTGAGGGCCACTCGAT‑GGTAGAGCGTATCATCATGAACTACATAGAGTCTAACAGTTACCGCGTGACTCTCTTTGAGGCTCTCAAACAGTTAGTCGTAGCTGGTAACGTCCTGCTGTACCTACCGGAACCGGAAGGGTCAAACTATAATCACATGAAGCTGTACCGATTGTCTTCTTATGTGGTCCAACGAGACGCATTCGGCAACGTTCTGCAAATGGTGACTCGTGACCAGATAGCTTTTGGTGCTCTCCCTTAGGACATCCGTAAGGCTGTAg        <  1:375376/300‑1 (MQ=255)`  
                                                                                                                                                                                                                                                                                                 `aCCCCGATGAACTCGCTAAGGTCGATGAGGGCCTCTCGAT‑GGTAGAGCGTATCATCATGAACTACATTGAGTCTAACAGTTACCGCGTGACTCTCTTTGAGGCTCTCAAACAGTTAGTCGTAGCTGGTAACGTCCTGCTGTACCTACCGGAACCGGAAGGGTCAAACTATAATCCCATGAAGTTGTACCGATTGTCTTCTTATGTGGTCCAACGAGACGCATTCGGCAACGTTCTGCAAATGGTGACTCGTGACCAGATAGCTTTTGGTGCTCTCCCTGAgctgtctcttatacacatctc       >  1:30286/1‑281 (MQ=255)`  
                                                                                                                                                                                                                                                                                                  `aagagacagACTGGCTAAGGTCGATGAGTGCCTCGCGAT‑GGTAGATCGTATCATCATGAACTAAATTGAGTCTAACATTTACCGCGTGACTCTCTTTGAGGCTCTCAAACAGTTAGTCGTAGCTGGTAACGTCCTGCTGTACCGACCGGAACCGGAAGGGTCAAACTATAATCCCATGAAGCTGTATCGATTGTCTTCTTATGTGGTCCAACGAGCCGCATTCGGCAACGTTCTGCAAATGGTGACTCGTGACCAGATAGCTTTTGGTGCTCTCCCTGAGGACATCCGTAAGGCTGTAGaa      <  1:71002/293‑1 (MQ=255)`  

                                                                                                                                                                                                                                                                                                          `|`                                                                                                                                                                                                                                                                                                           
`GAGAAACGAACAGGACTTGCGGAGGATGGCGCAAAGTCTGTCTATGAGCGTTTAAAGAACGACCGTGCTCCCTATGAGACACGCGCTCAGAATTGCGCTCAATATACCATCCCATCATTGTTCCCTAAGGACTCCGATAACGCCTCTACAGATTATCAAACTCCGTGGCAAGCCGTGGGCGCTCGTGGTCTGAACAATCTAGCCTCTAAGCTCATGCTGGCTCTATTCCCTATGCAGACTTGGATGCGACTTACTATATCTGAATATGAAGCAAAGCAGTTACTGAGCGACCCCGATGGACTCGCTAAGGTCGATGAGGGCCTCTCGAT‑GGTAGAGCGTATCATCATGAACTACATTGAGTCTAACAGTTACCGCGTGACTCTCTTTGAGGCTCTCAAACAGTTAGTCGTAGCTGGTAACGTCCTGCTGTACCTACCGGAACCGGAAGGGTCAAACTATAATCCCATGAAGCTGTACCGATTGTCTTCTTATGTGGTCCAACGAGACGCATTCGGCAACGTTCTGCAAATGGTGACTCGTGACCAGATAGCTTTTGGTGCTCTCCCTGAGGACATCCGTAAGGCTGTAGAAGGTC  >  NC_001604.1/20246‑20840`

  

Alignment Legend

Aligned base mismatch/match (shaded by quality score): `ATCG/ATCG` `< 3 ≤` `ATCG/ATCG` `< 10 ≤` `ATCG/ATCG` `< 19 ≤` `ATCG/ATCG` `< 34 ≤` `ATCG/ATCG` `< 38 ≤` `ATCG/ATCG`

Unaligned base: `atcg`    Masked matching base: `atcg`    Alignment gap: `‑`    Deleted base: `‑`
